# Solution‐Crystalized AgBiS_2_ Films for Solar Cells Generating a Photo‐Current Density Over 31 mA cm^−2^

**DOI:** 10.1002/advs.202406998

**Published:** 2024-10-09

**Authors:** Ludmila Cojocaru, Ajay Kumar Jena, Miwako Yamamiya, Youhei Numata, Masashi Ikegami, Tsutomu Miyasaka

**Affiliations:** ^1^ Toin University of Yokohama Kanagawa 225‐8503 Japan; ^2^ Komaba Institute for Science The University of Tokyo Tokyo 153‐8904 Japan

**Keywords:** AgBiS_2_ absorber, AgBiS_2_ solar cells, high current, simple solution coating, solution‐crystallized

## Abstract

In response to the toxic heavy metal absorbers in perovskite solar cells (PSCs), this work focuses on the development of an environmentally friendly simple solution‐processed infrared (IR) absorber. In this work, a simple solution‐crystallized IR‐absorbing AgBiS_2_ film is reported by spin‐coating silver, bismuth nitrates, and thiourea dissolved in dimethylformamide (DMF) to produce thick AgBiS_2_ film. Extensive optimization of the precursor concentrations thicknesses and conductive substrates used allow for obtaining 250 nm AgBiS_2_ film with different crystal sizes. When applied as an absorber in solar cells, solution‐crystalized AgBiS_2_ thick film delivers an extraordinarily high current density of over 31 mA cm^−2^. The devices show high stability under continuous 100 mW cm^−2^ illumination and when stored in the dark for more than six months. When the AgBiS_2_ layer is fabricated in a gradient fashion combining one layer of 0.25 m and three layers of 0.5 m precursor concentrations, the efficiency of 5.15% is registered which is the highest reported for the simple solution‐crystallized AgBiS_2_ films.

## Graphical abstract

In this work, a simple solution‐crystallized IR‐absorbing AgBiS_2_ film is reported by spin‐coating silver, bismuth nitrates, and thiourea dissolved in dimethylformamide to produce thick AgBiS_2_ film. When applied as an absorber in solar cells, solution‐crystalized AgBiS_2_ film delivered an extraordinarily high current density of over 31 mA cm^−2^.

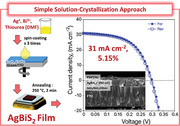

## 1 Introduction

The perovskite solar cell (PSC) has shown unprecedentedly rapid progress in power conversion efficiency (PCE) that has been improved to over 26% as a single junction and 33.9% in tandem with silicon solar cells.^[^ ^1^, ^2^ ^]^ Great efforts are being also put into the commercialization of the new technology. However, the current issues that are impeding the commercialization of lead‐based PSCs are their stability under operating conditions and the toxicity of lead.^[^ ^3^, ^4^, ^5^, ^6^ ^]^ Although in recent years many reports on improved stability of the PSCs while following the ISOS protocols^[^ ^6^ ^]^ have come out^[^ ^7^, ^8^ ^]^ it still falls short of the commercial requirements. Hence, stability is still a challenge while the other most critical issue is Pb‐toxicity. According to the European Union, RoHS directive, the maximum accepted weight concentration of lead in homogeneous materials is 1000 mg of lead per kg of the total material.^[^ ^9^ ^]^ To avoid lead, many works have been devoted to the development of lead‐free absorbers.^[^ ^10^ ^]^ Among all reported, although Sn‐based materials seem to have been most intensively investigated their stability remains worse than the Pb‐based counterparts. In comparison, Bi‐based absorbers like Ag_a_Bi_b_I_x_, and AgBiS_2_ having bandgap varying from 1.9 to 1.1 eV show better stability and therefore are more promising.^[^ ^11^, ^12^ ^]^ Bismuth has already been applied in photovoltaics for the interconnection of heterojunction solar cells due to its low melting point.^[^ ^13^ ^]^ Many cosmetics and pharmaceuticals also contain bismuth, which is approved by the US Food and Drug Administration.^[^ ^14^ ^]^ It is therefore interesting to further investigate Bi‐based photovoltaic materials for lower toxicity and lesser environmental impact.

Pure nanocrystalline AgBiS_2_ quantum dots (QD) without iodide ions in the structure have been extensively studied in the past.^[^ ^15^, ^16^, ^17^, ^18^, ^19^, ^20^ ^]^ The merits of QD‐AgBiS_2_ consist of its high absorption coefficient (10^5^–10^6^ cm^−1^), a bandgap ranging from 1.0 to 1.5 eV, and extremely stable material in the air.^[^ ^15^ ^]^ Generally, hot injection is a widely used method to synthesize high‐quality QD‐AgBiS_2_.^[^ ^16^, ^17^ ^]^ Efficiencies of solar cell cells with QD‐AgBiS_2_ reported in the literature are listed in Table S1 (Supporting Information). Such a QD‐synthesis approach, in general, sufficiently proves the great potential of AgBiS_2_ as a photovoltaic material but the method has disadvantages of low productivity and added complexity and cost to the overall fabrication of the solar cells. In this regard, developing a simple chemical approach to directly form AgBiS_2_ films on substrates could be advantageous and potentially accelerate the progress. However, to our surprise, we could not see much work with this approach except for three studies.^[^ ^21^, ^22^, ^23^ ^]^ For instance, Gu et al.^[^ ^22^ ^]^ studied the complexation among the precursor nitrate metal salts, bismuth nitrate (Bi(NO_3_)_3_), silver nitrate (Ag(NO_3_)), thiourea, and dimethyl sulfoxide (DMSO) and successfully made AgBiS_2_ films directly on the substrates but unfortunately, they did not implement them in solar cells as an absorber.^[^ ^23^ ^]^

As such simple methods have not been explored much in terms of their effect on film morphology, crystallinity, and eventually solar cell performance, in this work, we used a simple spin‐coating method and crystallized the precursor solution containing nitrate salts, AgNO_3_, Bi(NO_3_)_3_, and thiourea as a source of sulfur dissolved in DMF directly on substrates to form AgBiS_2_ films, and studied the influence of film characteristics on the solar cell performance. The thickness and morphology of the films were optimized through varying concentrations and multiple layers of deposition. A film of optimum thickness and morphology with poly((3‐hexylthiophene) (P3HT)) as hole transport layer (HTL) showed an impressive PCE of 5.15% with near to 100% retention of PCE after continuous irradiation for 1 h and for more than six months when the samples were stored in the dark. This is the highest efficiency for solution‐crystallized AgBiS_2_ solar cells reported so far based on a simple deposition method, demonstrating the great potential of AgBiS_2_ as a solution‐processible absorber for efficient, stable, and environmentally friendly solar cells.

## 2 Results and Discussion

### 2.1 Simple Solution‐Crystallized AgBiS_2_ Film Formation

**Figure 1:**
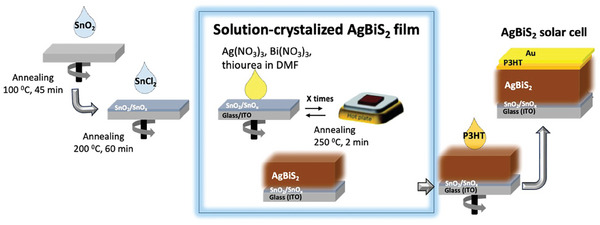
Fabrication steps of AgBiS_2_ solar cells.

**Figure** **1** shows the structure and preparation steps of AgBiS_2_ solar cells. Different from the methods followed for making AgBiS_2_ nanocrystal/QDs films, in this work, a simple one‐step deposition method using a precursor solution containing 0.5 m of AgNO_3_, 0.5 m of bismuth (III) nitrate pentahydrate Bi(NO_3_)_3_ *·*5H_2_O_,_ and 1.25 m thiourea as a source of sulfur was employed to form AgBiS_2_ film directly on the transparent conductive oxide (indium‐doped tin oxide glass In:SnO_2_, ITO or fluorine‐doped tin oxide F:SnO_2_, FTO) substrates. The powders were dissolved in DMF and the solution was spin‐coated on top of tin (IV) oxide (SnO_2_/SnO_x_) layers, annealed at 250 °C for 2 min to form a film with intense black color, which was confirmed to be AgBiS_2_ from X‐ray diffraction (XRD) (discussed later). Complexation happens between Bi(NO_3_)_3_ *·*5H_2_O and thiourea, and the latter also bonds to AgNO_3_ in the solution. During treatment at high temperatures, thiourea is decomposed releasing CS_2_ (along with HNCS, and NH_3_), which reacts with Ag and Bi to result in the AgBiS_2_ black film.^[^ ^22^, ^23^ ^]^ To increase film thickness the precursor solution was repeatedly coated while annealing each layer at 250 °C for 2 min and cooling down to room temperature before each layer. The effect of thickness and morphology on the cell performance was investigated (discussed in later sections).

It is known that the formation of a high‐quality perovskite layer depends on the environmental conditions.^[^ ^24^ ^]^ The humidity is a key factor controlling the crystal growth, morphology, and homogeneity of the perovskite film that in turn influences the efficiency of the solar cells. Therefore, we found that AgBiS_2_ film prepared at relative humidity (RH) <20% shows a porous film with agglomerated particles of size 71.3±33.1 nm (Figure S1a, Supporting Information). On the other hand, AgBiS_2_ films prepared at RH > 40% show particle sizes of 32.5±6.7 nm and compact homogeneous films (Figure S1a, Supporting Information). Additionally, when the film is prepared under RH<20% crystallinity becomes slightly better and peaks located at 27.5, 31.8, 45.5, and 53.9 correspond to (111), (200), (220), and (311) planes of cubic AgBiS_2_ (JCPDS number 89–3672), respectively (Figure S1b, Supporting Information). The XRD results are consistent with scanning electron microscopy (SEM) observations and both confirm that the crystallinity and morphology of AgBiS_2_ depend on the atmospheric conditions; larger agglomerated particles are formed at RH<20%.

The solar cells were fabricated with ITO/SnO_2_/SnO_x_/AgBiS_2_/P3HT/Au configuration and their photovoltaic performances were analyzed. P3HT was selected as an efficient HTL, with an electron affinity of 4.75 eV, and slightly tuned to the valence band of AgBiS_2_ (5.38 eV) by a shift to 4.82 eV after post‐thermal treatment at 110 °C for 10 min (Figure S2, Supporting Information). As shown in Figure S1c (Supporting Information) higher PCEs are obtained from the devices prepared in the dry atmospheric conditions. We observe a dramatic increase in all photovoltaic parameters (short‐circuit current density, J_sc;_ open‐circuit voltage, V_oc;_ and fill factor, FF) for the devices prepared in dry atmospheric conditions. These results suggest that the porous AgBiS_2_ film with bigger particle sizes works better than the compact film with smaller particle sizes. As a result, using dry atm conditions (RH<20%), a *PCE* of 2.18%, a *J_sc_* of 20.6 mA cm^−2^, a *V_oc_* of 0.258 V, and an *FF* of 0.41 were obtained (Table S2, Supporting Information). Based on these results, we hypothesize that the morphology and porosity of AgBiS_2_ could be one of the main factors contributing to high‐performance solar cells. The following optimizations for the fabrication of solar cells were performed in the dry room.

### 2.2 AgBiS_2_ Thickness Variation and Photovoltaic Response of the Solar Cells

**Figure 2:**
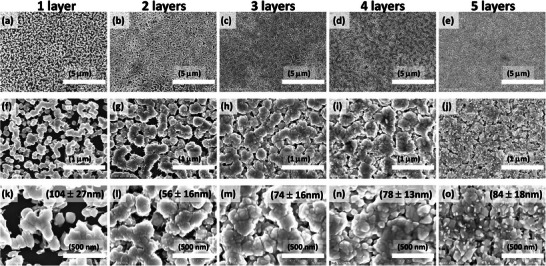
Surface SEM images of AgBiS_2_ films prepared layer‐by‐layers, a,f,k) 1 layer AgBiS_2_, b,g,l) 2 layers AgBiS_2_, c,h,m) 3 layers AgBiS_2_, d,i,n) 4 layers AgBiS_2_, e,j,o) 5 layers AgBiS_2_, a–e) 10k magnification AgBiS_2_, f–j) 50k magnification AgBiS_2_, k–o)100k magnification AgBiS_2._

**Figure 3:**
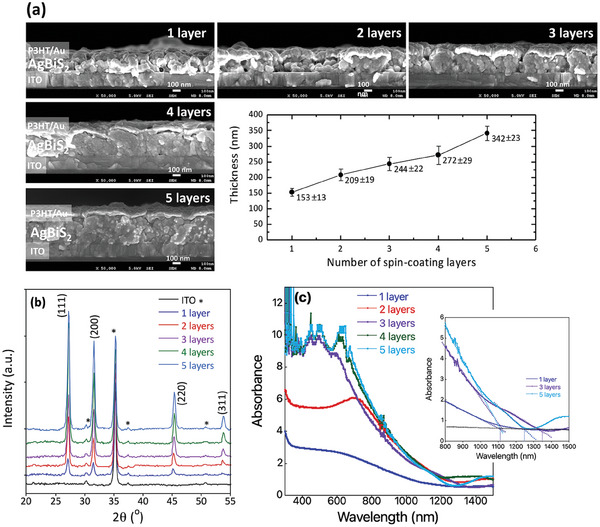
AgBiS_2_ solar cells prepared by using 0.5 m of AgNO_3_, 0.5 m of Bi(NO_3_)_3_ *·*5H_2_O_,_ and 1.25 m thiourea precursor, a) cross‐section SEM images of AgBiS_2_ solar cells with thickness variation, and mean values with standard deviation are presented, b) XRD pattern of AgBiS_2_ layer‐by‐layer coating, c) absorption spectra of AgBiS_2_ films prepared on ITO substrates.

To understand the relation between the film morphology and thickness, and the photovoltaic response of solar cells, AgBiS_2_ films were prepared by spin‐coating layer‐by‐layer up to five layers. **Figure** **2** displays the top SEM images of the AgBiS_2_ films (1, 2, 3, 4, and 5 layers) and **Figure** **3**a shows the cross‐sectional SEM images of corresponding devices. On repeating the spin‐coating process from 1 to 5 layers, the coverage is improved and the AgBiS_2_ film turns denser and more homogeneous (Figure 2a–e). In addition, it was interesting to observe that with multiple layers, the films progressively grew into a hierarchically structured morphology with large clusters composed of small particles. The small primary particles became more visible and their size increased from the 1st to the 5th layers, while coverage increased gradually. At the same time, the size of the secondary large particles also gradually increased. After the 1st layer, the particles of 104 ± 27 nm size with clusters size of 246 ± 126 nm were measured, however, after 2nd layer these clusters grew up to 418±133 nm while the primary particles of size 56±16 nm became visible. The size of these primary particles increased to 74±16 nm, 78±13 nm, and 84±18 nm after 3rd, 4th, and 5th layer deposition, respectively and the clusters grew up covering the whole surface of the substrate. As the number of spin‐coated layers is increased the small particles convert into bigger ones that can be attributed to the Ostwald ripening, that is dissolution of the smaller particles and agglomeration into larger ones over time, during the solvent drying at 250 °C. With multiple layer deposition, the added precursor to the already formed layer starts necking the primary particles, resulting in enlarged clusters, and at the same time, surface redissolution and recrystallization lead to the formation of the observed hierarchical morphology. A proposed mechanism of growth of such morphology is shown in Figure S3 (Supporting Information).

The film thickness seems to increase first slowly and then proportionally from 153±13 nm for the 1st layer to 342±23 nm for the 5th layer because of the progressive filling up of the pores in the films (Figure 3a). Hence, with the increase in the number of coatings, the porosity of the film decreases while more small particles are observed.

All the XRD peaks corresponded to the cubic AgBiS_2_, JCPDS number 89–3672 (Figure 3b). Although the morphology changed with the spin‐coating cycles the average crystallite size (24.4±1.72 nm), determined from XRD, did not change. The elemental composition of 1, 3, and 5 layers of AgBiS_2_ was analyzed by energy‐dispersive X‐ray spectroscopy, results are shown in Figure S4 (Supporting Information). Excluding Sn and O, which was coming from FTO/SnO_2_ substrate, the estimated atomic ratio of Ag:Bi:S for all the layers (inset in Figure S4, Supporting Information) was found to be ≈1:1:1.7, presenting slight sulfur deficiency in all the films. Hence, in general, the elemental analysis did not indicate any major impurity in the films.

**Figure 4:**
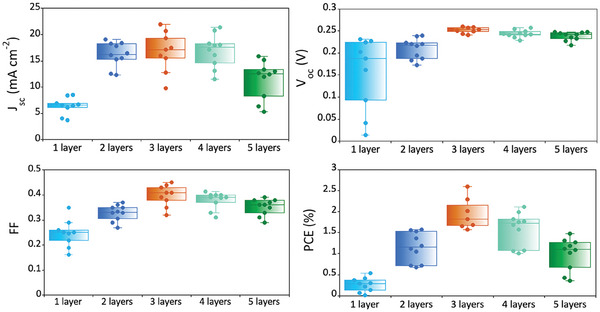
Photovoltaic parameters of the solar cells versus the thickness of AgBiS_2_ film.

The optical absorption spectrum extends to a far IR region with a slight difference in absorption edges for films made by different numbers of coating. The absorption gets saturated after 2nd layer and the optical absorption coefficient registered for all the AgBiS_2_ films (Figure S5a, Supporting Information) present values greater than 10^5^ cm^−1^ that is in good agreement with already reported works.^[^ ^17^, ^20^ ^]^ For all the layers, an absorption edge at ≈1100 nm corresponding to a bandgap of ≈1.12 eV (direct bandgap) is observed while an additional absorption edge at ≈1247 and 1350 nm (less than 1 eV) appears, which is clearer from the external quantum efficiency (EQE) results shown in below section (Figure 7b) and for the films made by three and more layers (Figure 3c). This can be related to the mixed particle size in the case of multiple layers. It is known that the bandgap of AgBiS_2_ decreases with an increase in particle size.^[^ ^25^ ^]^ In our case, with multiple‐layer coating primary particle size increases while small nanoparticles are also seen on the surface of large particles. Hence, we believe that the low band edge (≈0.9 eV) corresponds to large particles and 1.12 eV represents the bandgap of small particles. The position of the valence band of different AgBiS_2_ coatings is estimated to be similar to all films at 5.3 eV (Figure S5b, Supporting Information). As a result, the conduction band offset between low bandgap and high bandgap nanoparticles of AgBiS_2_ was beneficial for charge collection through gradient band structure as shown in Figure S6 (Supporting Information). Such gradient band structure was also recently reported by Konstantatos et al.^[^ ^25^ ^]^ The authors mixed small (4.7 nm) and large (7.1 nm) nanocrystals of AgBiS_2_ with higher and lower bandgaps respectively to intentionally create an energy band alignment that facilitated carrier collection and therefore exhibited an impressive efficiency of over 7% for QD‐AgBiS_2_ thick film solar cells. **Figure** **4** shows the photovoltaic parameters of the cells employing AgBiS_2_ films of different thickness and morphology. It can be seen that the *J_sc_, V_oc_*, and *FF* kept increasing to up to 3 layers (244±22 nm), then *J_sc_* and *FF* gradually decreased while *V_oc_* remained relatively stable.

After three layers, all the photovoltaic parameters dropped gradually as porosity decreased and thickness increased. Therefore, decreased performance after three layers could be due to either increased thickness or decreased porosity. To understand further, we tried to compare the above results with more compact films of similar thickness, which were made by varying the concentration of the precursor solution.

### 2.3 The Concentration of the Precursors, AgBiS_2_ Morphology, and Solar Cells’ Performances

**Figure 5:**
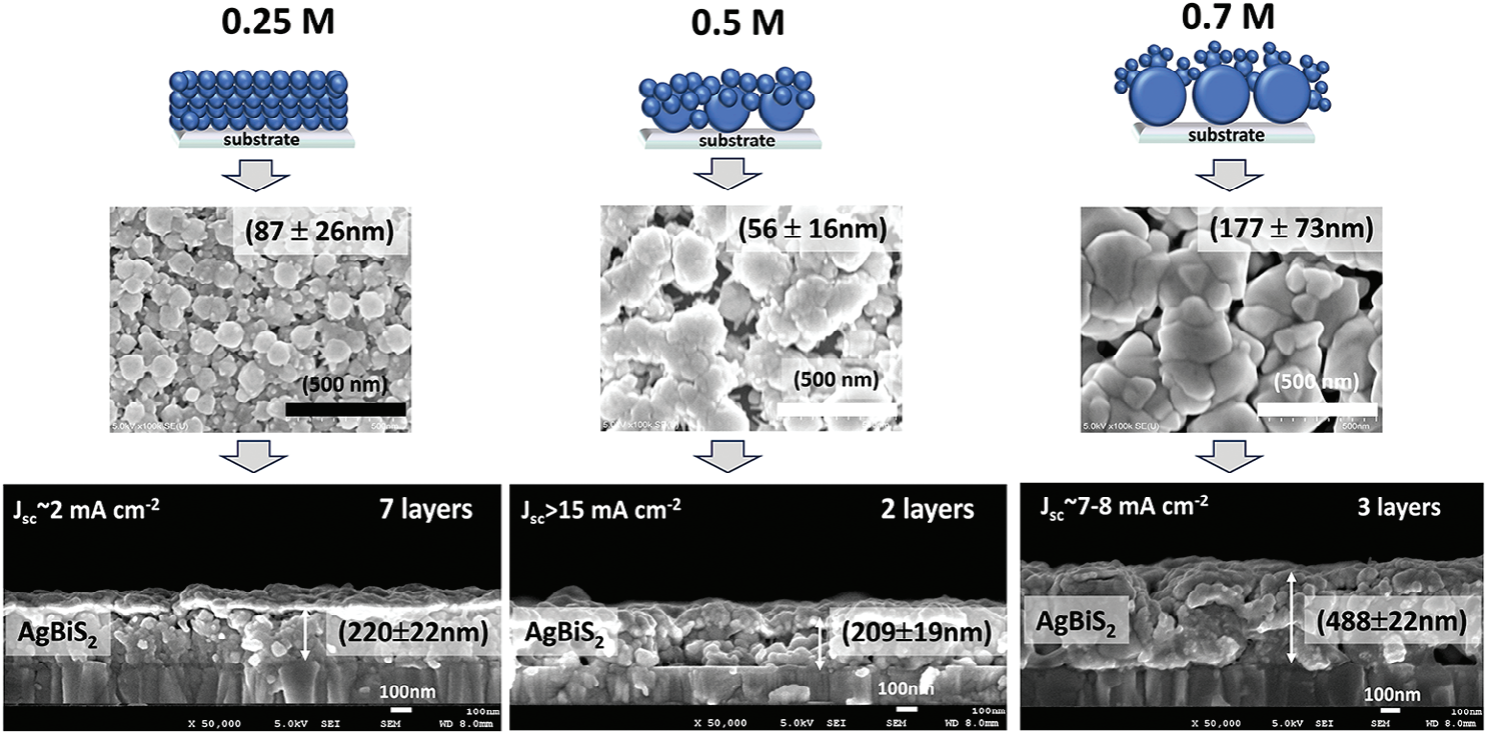
A schematic presentation of the crystal and morphology of AgBiS_2_ films versus concentration of the precursors.

The AgBiS_2_ films were prepared with two precursor concentrations, (*precursor 1* with low concentration: 0.25 m of AgNO_3_, 0.25 m of Bi(NO_3_)_3_·5H_2_O, and 0.6 m of thiourea, and, *precursor 2* with high concentration: 0.70 m of AgNO_3_, 0.70 m of Bi(NO_3_)_3_·5H_2_O, and 1.75 m of thiourea) other than the standard (0.5 m) discussed above. X‐ray diffraction patterns of those AgBiS_2_ films are shown in Figure S7(a,b) (Supporting Information). Similar to the 0.5 m case (Figure 3b) four main characteristic peaks for (111), (200), (220), and (311) planes were detected for all samples, with the increase in the intensity of all the peaks with an increase in thickness. Hence, it confirmed that there was no difference in the crystal structure of the AgBiS_2_ made from 0.25, 0.5, or 0.7 m solutions except a slight difference in crystallite size, which was 9.6 ± 1.9 nm for *precursor 1* and 25.5 ± 2.5 nm for *precursor 2*. However, there was a clear difference in the morphology (Figure S8, Supporting Information) of the films, which can be attributed to the crystallization process that changes with precursor concentration. The cross‐sectional SEM images (Figure S9, Supporting Information) show that the absorber film made from *precursor 1* (0.25 m) is denser with smaller particles than the ones made from 0.5 m solution, even for the 1‐layer coated film with lower thickness (102 ± 12 nm). The porosity of AgBiS_2_ film decreases and thickness increases for subsequent layers. On the other hand, the film made from *precursor 2* (0.7 m) was found to be thicker but more porous than the standard (0.5 m). Average thicknesses of 102 ± 12, 124 ± 21, 158 ± 8, 188 ± 15, and 220 ± 22 nm were measured for 3, 4, 5, and 6 layers of deposition of *precursor 1*, respectively. The *precursor 2* forms films with an average thickness of 390 ± 27, 413 ± 29, 488 ± 22, and 551 ± 44 nm for 1, 2, 3, and 4 layers of deposition, respectively (Figure S9, Supporting Information). Hence, it was clear that the particle size, thickness, and porosity of the films increased with increasing precursor concentration from 0.25  to 0.70 m. However, it was more interesting to see the difference in morphology than the thickness, which seemed to influence the cell performance dramatically. As can be seen in Figure S10 (Supporting Information), decreasing the concentration of the precursor from standard 0.5 to 0.25 m (*precursor 1*) leads to a dramatic decrease in device performance. The *J_sc_, V_oc_*, and *FF* of all the cells made from *precursor 1* were way lower than the 0.5 m case and they decreased with the number of layers. The film made by seven layers deposition of 0.25 m solution that had a thickness (220 ± 22 nm) similar to the two layers film made from 0.5 m solution (209 ± 19 nm), had more uniform particles and was different from the hierarchical morphology seen in the 0.5 m case. Although the former looked denser with smaller pores, it seems that the particles are not electronically well connected (poor necking) resulting in poor carrier transport. Thus, while the *J_sc_* obtained from a more porous film of a thickness of 209 ± 19 nm but with a hierarchical morphology (two layers, 0.5 m) was >15 mA cm^−2^ (*PCE* of 1%) that for the denser film without hierarchical morphology (seven layers, 0.25 m) was only ≈2 mA cm^−2^ (*PCE* < 0.1%). Moreover, even the thinnest film (102 ± 12) made from 0.25 m solution yielded a current density of only 6 mA cm^−2^. This clearly indicated that certain porosity and the hierarchical morphology with better interparticle connectivity were more critical for cell performance. Indeed, this was also observed for AgBiS_2_ films made in dry and humid conditions (Figure S1, Supporting Information). The film made under humid conditions had a morphology similar to 0.25 m case, with smaller pores (denser) but poor interparticle connectivity leading to no photocurrent at all. For the higher concentration case (0.7 m), *J_sc_* increased with thickness and it was interesting to observe that the thickest film (three layers, 488 ± 22 nm) that showed a *J_sc_* of 7–8 mA cm^−2^ had a similar hierarchical morphology with small primary particles on the large secondary particles as seen in the 0.5 m case but with slight change in particle size and shapes. Therefore, we believe this hierarchical morphology, as depicted in the schematic in **Figure** **5**, is the main reason for higher *J_sc_* and *PCE*.

### 2.4 The Best‐Performing AgBiS_2_ Solar Cells, Stability, and Recombination Losses Analysis

**Figure 6:**
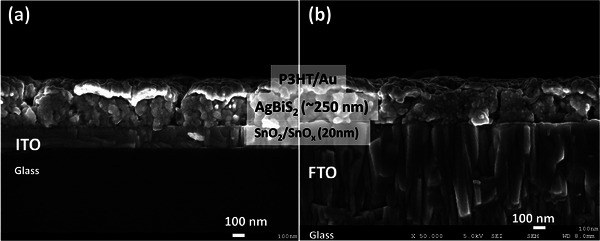
Cross‐section SEM view of the best‐performing AgBiS_2_ solar cells, a) AgBiS_2_ film prepared on ITO, b) AgBiS_2_ film prepared on FTO.

As AgBiS_2_ solar cells prepared using 0.5 m precursor concentration exhibited the best overall performance, our further investigations were focused on this device processed on different substrates (ITO, and FTO). Based on the optical measurements depicted in Figure S11 (Supporting Information), it can be observed that the transmittance in the infrared region (1100‐1400 nm) of FTO is higher than ITO. This high transmittance can potentially increase the number of photons entering the AgBiS_2_ absorber layer. Hence, we checked the performance of cells prepared on FTO substrates (**Figure** **6**). First, the morphology of the AgBiS_2_ films prepared on ITO and FTO by the optimum fabrication process (i.e., 0.5 m, three layers, post‐annealing at 110 °C for 10 min under dry atm conditions) was checked. No difference in the morphology of the AgBiS_2_ films was observed, and the porosity and the film thickness were similar in both cases (Figure 6a,b).

**Figure 7:**
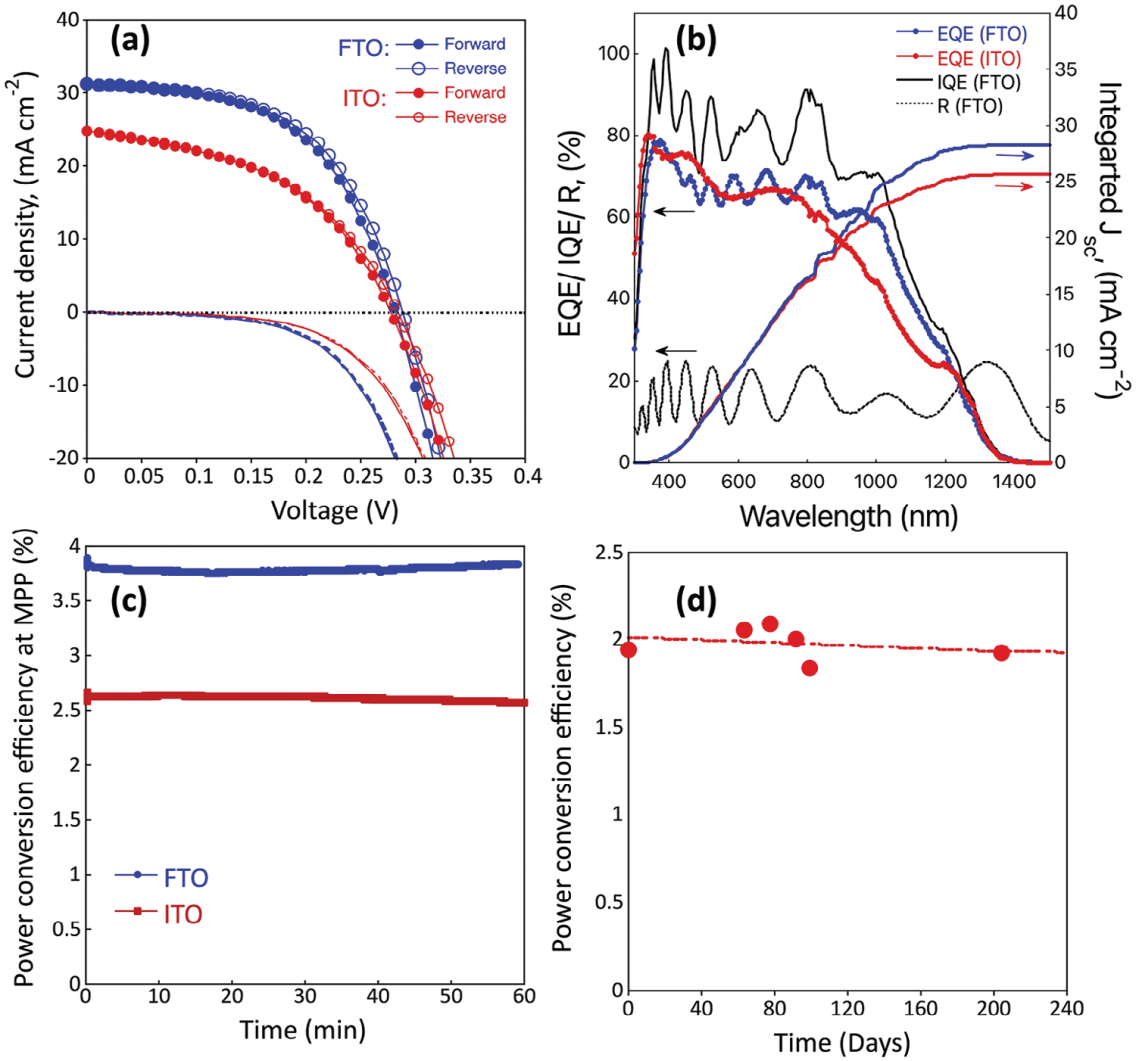
The best‐performing AgBiS_2_ solar cells prepared on FTO and ITO substrates, a) *I–V* curves with reverse (open dots) and forward (full dots) scans, b) EQE, IQE, of solar cell and reflectance of AgBiS_2_ coated on FTO/SnO_2_ substrates, and integrated current from EQE c) PCE at the maximum power point over time, measured under continuous 100 mW cm^−2^ irradiation, and controlled atmosphere (RH<0%, temperature of 20 °C), d) AgBiS_2_ prepared on ITO, solar cells kept in dry and dark conditions, and PCE measured on different days of storage.

The FTO substrate‐based devices yielded higher efficiencies than those on ITO substrates (**Figure** **7**a). On the reverse (forward) scan, *PCE* of 4.85 (4.73) %, *V_oc_* of 0.288 (0.281) V, *J_sc_* of 31.2 (31.2) mA cm^−2^, and *FF* of 0.54 (0.54) were registered for FTO substrates (**Table** **1**).

**Table 1:** Photovoltaic parameters of the AgBiS_2_ solar cells, AgBiS_2_ film prepared on FTO and ITO substrates.

Substrates	^Integrated^ J_sc_, mA cm^−2^ ^a)^	J_sc_, mA cm^−2^ ^b)^	V_oc_, V	FF	PCE, %
FTO	28.2	31.2 (31.2)	0.288 (0.281)	0.54 (0.54)	4.85 (4.73)
ITO	25.6	24.9 (24.9)	0.281 (0.275)	0.46 (0.46)	3.22 (3.15)

^a)^ Note: ^*^Integrated *J_sc_* extracted from EQE measurements;

^b)^ photovoltaic parameters extracted from reverse(forward) I‐V curves.

The solar cells prepared on ITO exhibited a *PCE* of 3.22 (3.15) %, *V_oc_* of 0.281 (0.275) V, *J_sc_* of 24.9 (24.9) mA cm^−2^, and *FF* of 0.46 (0.46). The EQE spectrum illustrates high quantum efficiency leading to an integrated current of 28.2 and 25.6 mA cm^−2^ for the AgBiS_2_ solar cells prepared on FTO and ITO, respectively, which are in good agreement with the current from current‐voltage (*I–V)* measurements (Figure 7b). The small discrepancy of *J_sc_* extracted from *I*‐*V* curves EQE is widely observed in the perovskite and organic solar cells.^[^ ^26^, ^27^, ^28^ ^]^ This can be explained by different measurement setups and calibration methods used for *I*‐*V* and EQE. In addition, the discrepancy can depend on the choice of electrode materials, absorber composition, and deposition technique used.^[^ ^29^ ^]^

In particular, the *J_sc_* increased from 24.9 (ITO) to 31.2 (FTO) mA cm^−2^, presenting the highest current density reported so far for thick AgBiS_2_ film solar cells. The difference in *J_sc_* in the case of FTO compared with ITO can be related to the substrate transmittance that is higher in the near IR region for FTO as suggested by Hayase et al.^[^ ^30^ ^]^ The near IR region is also beneficial for the AgBiS_2_ due to its broad absorption spectrum that allows the conversion of more photons into electrons resulting in a high *J_sc_* of the devices. More importantly, the unencapsulated devices retained 98% of their initial efficiencies after 60 min under continuous operation at 100 mW cm^−2^ light irradiation (Figure 7c,d) and 95% of their initial *PCE* after 204 days (more than 6 months) of storage in the dark. Moreover, the high internal quantum efficiency (IQE) of the AgBiS_2_ on FTO, calculated from the ratio of the EQE and reflectance absorption of AgBiS_2_/SnO_2_/FTO substrates, indicates that the absorbed photons are quite efficiently converted to the charges.

**Figure 8:**
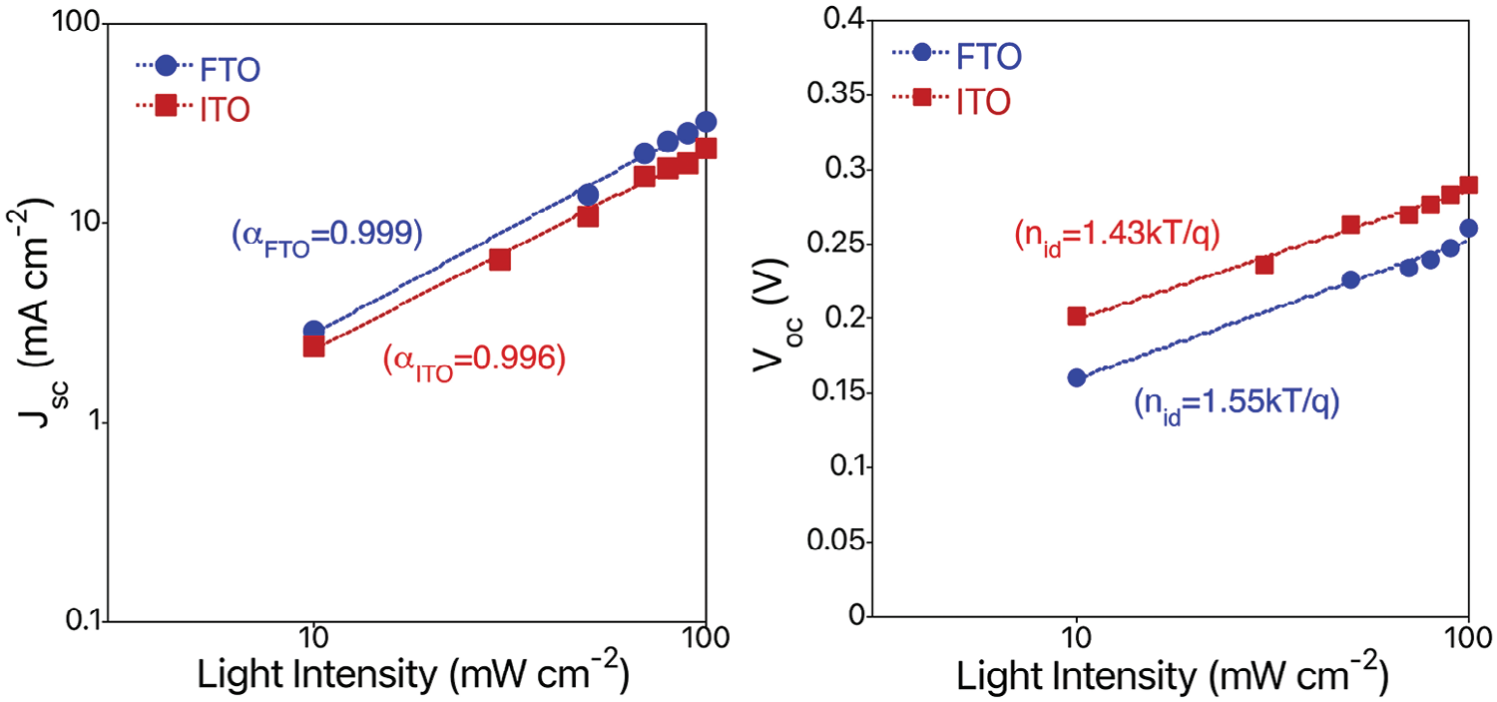
Current density (*J_sc_*) with a slope and open circuit voltage (*V_oc_*) with ideal factor, *n_id_* at different light intensities of AgBiS_2_ solar cells prepared on FTO and ITO substrates.

The light intensity dependence of *J_sc_* and *V_oc_* were investigated to assess the recombination losses in the solar cells prepared on FTO and ITO substrates. **Figure** **8** displays the intensity dependence of *J_sc_* and *V_oc_* for AgBiS_2_ solar cells, based on the *I–V* curves at different light intensities (Figure S12, Supporting Information). In the power law dependence of *J_sc_* versus light intensity, *I*, described by *J_sc_* = *J*_0*sc*_  × *I* ^α^ *, α* close to unity suggests little involvement of bimolecular recombination.^[^ ^31^, ^32^, ^33^ ^]^ The *V_oc_*‐light intensity characteristics provide information on trap‐assisted recombination as estimated by the ideality factor (*n_id_*), $n_{id}=\frac{q}{k_{b}\times T}\;\times \frac{dV_{oc}}{d(\ln(I))}$, where *k_b_* is the Boltzmann constant, *T* is the temperature, *q* is the electron charge, and *I* is light intensity.^[^ ^34^ ^]^ Here, a high *n_id_* value (>1) implies carrier loss occurring predominantly via trap‐assisted recombination. In our device, *α * of 0.996 and 0.999 and n_id_ of *1.55* and *1.43* were obtained for the AgBiS_2_ prepared on ITO and FTO, respectively. These results imply that a large carrier loss occurring in the AgBiS_2_ device is substantially due to trap‐assisted recombination, which is slightly weaker for the solar cell prepared on FTO and the FTO‐based cells exhibit larger *J_sc_*, *V_oc_, FF*, and *PCE* than the solar cells prepared on ITO substrates. Moreover, we observed, no photoluminescence (PL) emissions from AgBiS_2_ film while a very weak peak at ≈600 nm was confirmed to be coming from the glass substrate, as observed for several other absorber layers such us Ag_2_BiI_5_, Cu_2_AgBiI_6_, Cs_2_AgBiI_6_. In fact, we believe this is the reason why all the reports so far, to the best of our knowledge, have not shown any PL results.^[^ ^35^ ^]^ Hence, no PL from the AgBiS_2_ films (Figure S13, Supporting Information) indicates high trap‐assisted recombination that may be related to imperfections in the purity of the crystal structure and/or inter‐particle connections, quenching luminescence completely.

**Figure 9:**
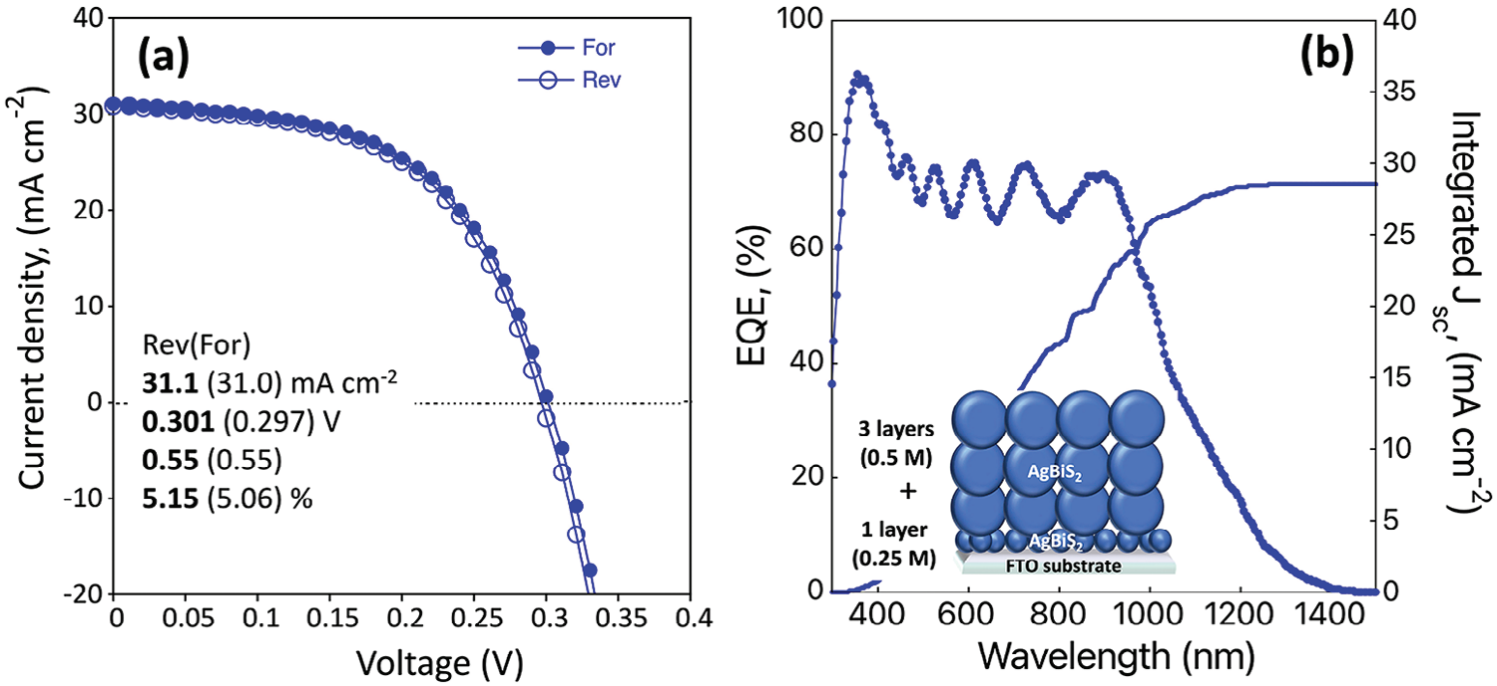
AgBiS_2_ solar cells of 1 layer of *precursor 1* and 3 layers of standard *precursor* solution containing 0.5 m of AgNO_3_, 0.5 m of Bi(NO_3_)_3_·5H_2_O, and 1.25 m of thiourea in DMF precursor solution, a) *I–V* curves with reverse (open dots) and forward (full dots) scans, b) EQE and integrated current, *J_sc_*.

Based on our observations on morphology evolution with different precursor concentrations, we made devices by combining 1 layer of precursor solution with a concentration of 0.25 m with three layers of precursor solution of 0.5 m (**Figure** **9**) to reduce the interfacial recombination loss, if any due to porous film of AgBiS_2_ at the electron transport layer. As expected, we observed an increase in *V_oc_* and a slight increase in *FF* when combining two different precursor solutions. The thick AgBiS_2_ solar cells delivered a *J_sc_* of 31 mA cm^−2^ that was in good match with an integrated *J_sc_* calculated from EQE. As a result, the *PCE* jumped from 4.85% for 3 layers (0.5 m) of precursor solution to 5.15% for the combination of 1 layer (0.25 m) and 3 layers (0.5 m) precursor solutions.

EQE was as high as 90% at ≈380 nm (Figure 9b) that was confirmed to follow the absorption of the AgBiS_2_ on the FTO/SnO_2_ substrates (Figure S14c, Supporting Information). Although a bare FTO shows a lower transmittance, 80% (at 380 nm, Figure S14a, Supporting Information), due to light scattering and reflection loss at the air‐FTO interface, the absorption for the FTO/SnO₂/AgBiS₂ sample was estimated to be close to 90% over the range of 300–600 nm (Figure S14c, Supporting Information). EQE showed a decrease at longer wavelengths, indicating an increase in carrier recombination loss.

## 3 Conclusion

Solution‐crystallized AgBiS_2_ films were prepared using one‐step spin‐coating method by varying the concentration of the precursors, number of coatings, and type of conductive substrates, resulting in AgBiS_2_ with different particle sizes and film morphologies. The deposition conditions were optimized and it was identified that the concentration of the precursor defined the growth of the particles on the conductive substrates and influenced the morphology of the film significantly. In general, a solution of lower concentration produces films with smaller particles but lesser porosity while a higher concentration solution results in larger particles and greater porosity. A low‐concentration precursor solution with 0.25 m of the nitrates salts, noted as *precursor 1*, resulted in an average particle size of 54 ± 21 nm for three layers and increased to 99 ± 44 nm for six layers, resulting in compact layer‐by‐layer crystalline AgBiS_2_ film. At a high precursor concentration (0.7 m) noted as *precursor 2*, the particle size of 132 ± 32 nm was observed for one layer of coating, and it increased linearly to 171 ± 44 nm for four layers of coating. However, in both low and high‐concentration cases, the performance of the solar cells was found to be low, 0.2% and 0.4%, respectively in the best cases. However, significant improvements in photovoltaic response were obtained using the precursor solution of 0.5 m of the nitrate salts. This can be attributed to a special hierarchical morphology consisting of small and large particles, and certain porosity in the film. An AgBiS_2_ film of such morphology and 250 nm thickness in combination with FTO/SnO_2_/SnO_x_ and P3HT could deliver an extraordinarily high current density of 31.2 mA cm^−2^, a power conversion efficiency of 4.85%. The cells showed high stability under continuous 100 mW cm^−2^ illumination, showing almost no change in performance after 1 h of operation, and on storage in the dark for more than seven months, with a slight decrease in performance. By combining one layer of 0.25 m with three layers of 0.5 m precursor solutions, a PCE of 5.15% was obtained. The results provide new approaches for simple solution‐crystallized AgBiS_2_ film that can work with reasonably high efficiency, close to many reported for quantum dots‐based cells, and more importantly the photocurrent density of 31 mA cm^−2^ that is the highest reported so far. These results endorse the great potential of AgBiS_2_ as a solution‐processible absorber for low‐cost, efficient, stable, and environmentally friendly solar cells. We believe that further optimization of morphology, and use of suitable electron transport layer and HTL can leap the performance to higher levels.

## 4 Experimental Section

### Materials

For conductive substrates, ITO, ≤10 Ω/sq was purchased from Geomatec Co., and FTO, ≤7 Ω/sq was purchased from SPD Lab Inc. SnO_2_, 15% in H_2_O colloidal dispersion was purchased from Alfa Aesar. Tin (II) Chloride (99.9%) was purchased from Fujifilm Wako Pure Chemical Corporation. DMF, 99.8% was purchased from Fujifilm Wako Pure Chemical Corporation. After several optimization tests, the precursor materials to prepare AgBiS_2_ were selected and AgNO_3_ was pursued from Sigma–Aldrich, Bi(NO_3_)_3_ *·*5H_2_O was from Wako Pure Chemical, and thiourea was from Sigma–Aldrich. Regio‐regular P3HT from Sigma–Aldrich with a molecular weight average of 50 000–100 000 dissolved in chlorobenzene (Aldrich) was used as HTL. All the materials were used as received, without any further purification.

### Solar Cells Fabrication

ITO and FTO substrates were washed successively with isopropanol/water (volume ratio of 1/1) and acetone for one hour in an ultrasonic bath. The dried conductive substrates were treated with UV‐Ozone (UV‐O_3_) cleaner for 10 min before deposition of the next layer. Immediately after UV‐O_3_ cleaning, the SnO_2_ and SnO_x_ layers were deposited following the procedure developed by Guo et al.^[^ ^36^ ^]^

AgBiS_2_ precursor solution containing AgNO_3_, Bi(NO_3_)_3_·5H_2_O, and thiourea was prepared in DMF by stirring the mixture at room temperature for ≈30 min. Based on the stoichiometric ratio of Ag:Bi:S (1:1:2) in AgBiS_2_, the thiourea as a source of sulfur was used in slight excess (i.e., Ag:Bi:S = 1:1:2.5). For precursors of different concentrations (0.25, 0.5, and 0.7 m), the concentration of AgNO_3_ and Bi(NO_3_)_3_ *·*5H_2_O were kept 0.25, 0.5 and 0.7 m while thiourea was varied accordingly to keep the Ag:Bi:S ratio 1:1:2.5 constant. A 150 mL precursor solution was deposited on the SnO_2_/SnO_x_ layers via spin‐coating at 4000 rpm for 50 s. After the spin‐coating, the film was annealed at 250 °C for 2 min on a hot plate. After that, the samples with a black film of AgBiS_2_ were collected from a hot plate and cooled down to room temperature for over 2 min. To prepare thicker AgBiS_2_ film, the precursor solution was repeatedly spin‐coated and annealed at 250 °C for 2 min for each layer. The P3HT layer was deposited on the AgBiS_2_ film by spin‐coating the P3HT solution (10 mg mL^−1^, in chlorobenzene, no dopants) at 0 rpm for 10s followed by 3000 rpm for 30s. Finally, an 80 nm Au electrode was deposited by thermal evaporation. To improve the contact between P3HT and Au the complete devices were annealed at 110 °C for 10 min.^[^ ^37^ ^]^ The precursor solutions preparation and spin‐coating of all layers including SnO_2_, AgBiS_2_, P3HT, etc. were done in an air ambient lab (no glove box) where the humidity was controlled at ≈20% relative humidity and a temperature of 20 °C.

### Film and Device Characterization

A scanning electron microscope (SU8000, Hitachi High‐Technologies Co. and JSM‐7500FA, Jeol) was used for checking the surface and cross‐sectional micrographs of AgBiS_2_ films and solar cells. The UV–vis absorption spectra of the films were acquired by using a Shimadzu UV‐1800 spectrophotometer. The highest occupied molecular orbital levels of P3HT and working potential (valence band) of AgBiS_2_ were estimated by photoelectron spectroscopy in the air using a photoelectron spectrometer (AC‐3E, Riken Keiki, Japan). The element composition was measured using an X‐ray fluorescence spectrometer (JSM‐7800F, JEOL, Japan).

The *I*‐*V* characteristics for reverse and forward scans of solar cells presented in Figures S1c, S10, S12 (Supporting Information) and Figures 4 and 8 were measured with the PEC‐L01 (Peccell Technologies) equipped with Xe lamp (BSOX150, BUNKOKEIKI). The light intensity was calibrated with a Si reference cell (BS‐520 BK, S/N536, BUNKOKEIKI). The conditions for *I–V* curves were set as, the starting voltage of 0.4 V, the end voltage of −0.1 V, the step voltage of 0.01 V, and scan speed of ≈50 mV s^−1^, and light soaking 1 min, with no bias voltage applied before measurement. The solar cells were measured with PEC‐I01 in the air in ambient atmospheric conditions.

The *I–V* curves with reverse and forward shown in Figures 7 and 9 were measured with the CEP‐2000 (BUNKOKEIKI) solar simulator equipped with Xe lamp (BSX150LC, BUNKOKEIKI). The light intensity was calibrated with a Si reference cell (BS‐520 BK, BUNKOKEIKI). The *I–V* curves were measured from a starting voltage of 0.4 V to an end voltage of −0.01 V at a scan speed of roughly 50 mV s^−1^. The solar cells were measured with CEP‐2000 in the air and controlled dry conditions (RH <0, temperature of 20 °C). The contact and active area of the cells were similar at 0.09 cm^2^.

Stability tests shown in Figure 7c were performed using the light source of CEP‐2000 and tracking the maximum power of solar cells continuously by PV Analyser VK‐PA‐100 (SPD Lab Inc.). The continuous light irradiation of 100 mA cm^−2^ in a controlled atmosphere (RH<0%, temperature of 20 °C) was used for stability tests. Stability tests under dark were done with the PEC‐L01 by measuring the *I*‐*V* curves of solar cells at different intervals. The samples were kept in the dark in a dry room (RH <20%).

The EQE (Figures 7 and 9) was measured from 300 to 1500 nm under a constant photon flux, using a set‐up (BUNKOKEIKI CEP‐2000) with a 150 W Xe arc lamp equipped with a monochromator.

## Conflict of Interest

The authors declare no conflict of interest.

## Author Contributions

L.C. and A.K. conducted the experiments; M.Y. characterized the morphology of AgBiS_2_ film; Y.N. validation, T. M. and M.I. supervised the project and funding acquisition. L.C., A. K., wrote the manuscript. All authors provided feedback and comments revising the manuscript.

## Supporting information



Supporting Information

## Data Availability

The data that support the findings of this study are available from the corresponding author upon reasonable request.
