# Supplementary material for: Solution‐Crystalized AgBiS2 Films for Solar Cells Generating a Photo‐Current Density Over 31 mA cm−2
Source: Adv Sci (Weinh). 2024 Oct 9;11(44):2406998. doi: 10.1002/advs.202406998 (PMC11600239; doi:10.1002/advs.202406998)
Supplement: Supplementary file 1 — Supporting Information [file ADVS-11-2406998-s001.docx]

SUPPORTING INFO

**Solution-Crystalized AgBiS_2_ Films for Solar Cells Generating a Photo-Current Density Over 31 mA cm^-2^**

*Ludmila Cojocaru,^1,2,^* Ajay Kumar Jena,^2^ Miwako Yamamiya,^1^ Youhei Numata,^1^ Masashi Ikegami,^1^*

*Tsutomu Miyasaka^1,^**

^1^Toin University of Yokohama, Kanagawa 225-8503, Japan

^2^Komaba Institute for Science, The University of Tokyo, Tokyo 153-8904, Japan

*Correspondence: [cojocaru@g.ecc.u-tokyo.ac.jp](mailto:cojocaru@g.ecc.u-tokyo.ac.jp), miyasaka@toin.ac.jp

**Table S1.** Efficiencies of solar cells with QD-AgBiS_2_ reported in the literature and our solution-crystallized IR-absorber AgBiS_2_ films.

| Device structure | AgBiS_2_ thickness, nm | J_sc_,  mA cm^-2^ | V_oc_,  V | FF,  % | PCE, % | Ref. |
| --- | --- | --- | --- | --- | --- | --- |
|  |  |  |  |  |  |  |
| ***Quantum Dots QD-AgBiS_2_*** |  |  |  |  |  |  |
| ITO/ZnO_2_/AgBiS_2_ /PTB7/MoO_3_/Ag | 37 | 22.1 | 0.45 | 63 | 6.31 | ^[[1]](#endnote-1)^ |
| ITO/ZnO/AgBiS_2_ /P3HT/Ag |  | 15.1 | 0.46 | 57 | 3.99 |  |
| ITO/ZnO_2_/AgBiS_2_/spiro-OMeTAD/MoO_3_/Ag |  | 6.6 | 0.42 | 42 | 1.16 |  |
| ITO/SnO_2_/AgBiS_2_/PTAA/MoO_3_/Ag | 30 | 27.11 | 0.495 | 68.4 | 9.17 | ^[[2]](#endnote-2)^ |
|  | 200 | 16.5 | 0.34 | 43 | 2.39 |  |
| ITO/ZnO/AgBiS_2_/spiro-OMeTAD/MoO_3_/Ag | ~70 | 18.1 | 0.241 | 35 | 1.5 | ^[[3]](#endnote-3)^ |
| ITO/ZnO-NP/ZnO-NW/AgBiS_2_/P3HT/Au | 420 (320+100) | 22.21 | 0.41 | 60 | 5.41 | ^[[4]](#endnote-4)^ |
| ITO/ZnO/AgBiS_2_/P3HT/MoO_3_/Al | ~180 | 16.56 | 0.23 | 36.75 | 1.40 | ^[[5]](#endnote-5)^ |
| ***Solution-crystalized AgBiS_2_ film*** |  |  |  |  |  |  |
| FTO/SnO_2_/SnO_x_/AgBiS_2_ film/P3HT/Au | ~250 | 31.1 | 0.301 | 55 | 5.15 | this work |


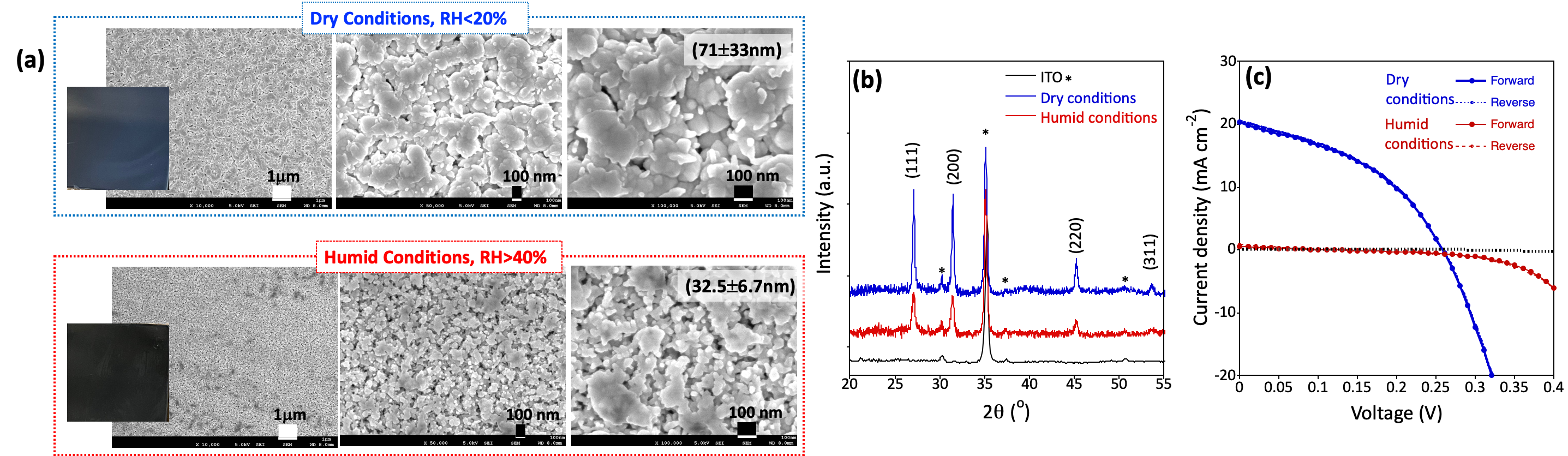
**Figure S1.** Morphology and photovoltaic performances of AgBiS_2_ (a) surface SEM images, (b) XRD pattern of the AgBiS_2_ films, (c) I-V curves for the AgBiS_2_ solar cells prepared in controlled dry (RH<20%) and normal atmospheric conditions (RH>40%).

**Table S2.** Photovoltaic parameters of the AgBiS_2_ solar cells, AgBiS_2_ film prepared in dry (RH<20%) and in humid (RH>40%) atmospheric conditions.

| Atmospheric conditions | J_sc_, mA cm^-2a)^ | V_oc_, V | FF | PCE, % |
| --- | --- | --- | --- | --- |
| RH<20% | 20.6 (20.3) | 0.258 (0.258) | 0.41 (0.41) | 2.18 (2.15) |
| RH>40% | 0.84 (0.61) | 0.127 (0.115) | 0.19 (0.19) | 0.02 (0.013) |

a)Note: Reverse (Forward) scans

**Figure S2.** Photoemission spectroscopy measurement (valence band position) of AgBiS_2_ and HOMO level of P3HT before/after annealing.

**Figure S3.** Schematic representation of the AgBiS_2_ film formation after coating layer-by-layer precursor solution, 1, 2, 3, 4, and 5 layers of AgBiS_2_.

**

**Figure S4**. EDX spectrum of the 1 layer, 3 layers, and 5 layers of AgBiS_2_ on FTO substrates.


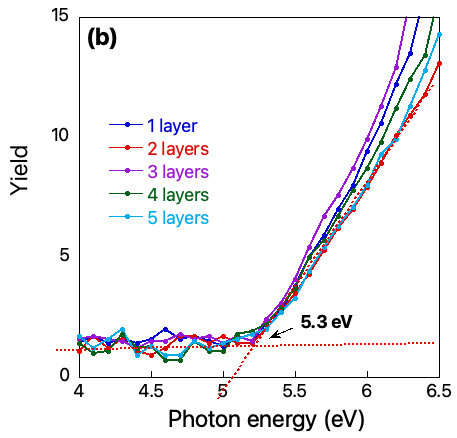


**Figure S5.** (a) Absorption coefficient and (b) photoemission spectroscopy measurement (valence band position) of AgBiS_2_ films with different thicknesses.


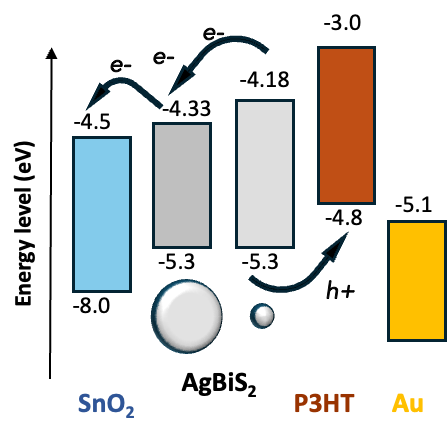


**Figure S6.** The gradient energy level diagram of different sizes AgBiS_2_.

**Figure S7.** XRD pattern of AgBiS_2_ film prepared layer-by-layer, using (a) *Precursor 1* and (b) *Precursor 2*.

*Precursor 1:* 0.25 M of AgNO_3_, 0.25 M of Bi(NO_3_)_3_·5H_2_O, and 0.6 M of thiourea

*Precursor 2:* 0.70 M of AgNO_3_, 0.70 M of Bi(NO_3_)_3_·5H_2_O, and 1.75 M of thiourea

**Figure S8.** Surface SEM images of AgBiS_2_ films prepared using *Precursor 1* (top) and *Precursor 2* (bottom) layer-by-layers at different magnifications (10k magnification, a-d; 50k magnification f-i; and 100k magnification k-n).

*Precursor 1:* 0.25 M of AgNO_3_, 0.25 M of Bi(NO_3_)_3_·5H_2_O, and 0.6 M of thiourea

*Precursor 2:* 0.70 M of AgNO_3_, 0.70 M of Bi(NO_3_)_3_·5H_2_O, and 1.75 M of thiourea

**Figure S9.** Cross-section SEM images of AgBiS_2_ solar cells with different AgBiS_2_ thicknesses, using *Precursor 1* (top images) and *Precursor 2* (bottom images)*.*

*Precursor 1:* 0.25 M of AgNO_3_, 0.25 M of Bi(NO_3_)_3_·5H_2_O, and 0.6 M of thiourea


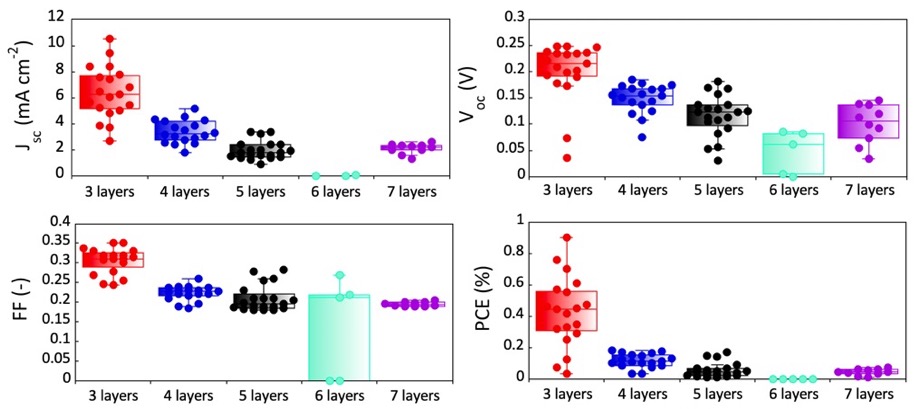
*Precursor 2:* 0.70 M of AgNO_3_, 0.70 M of Bi(NO_3_)_3_·5H_2_O, and 1.75 M of thiourea


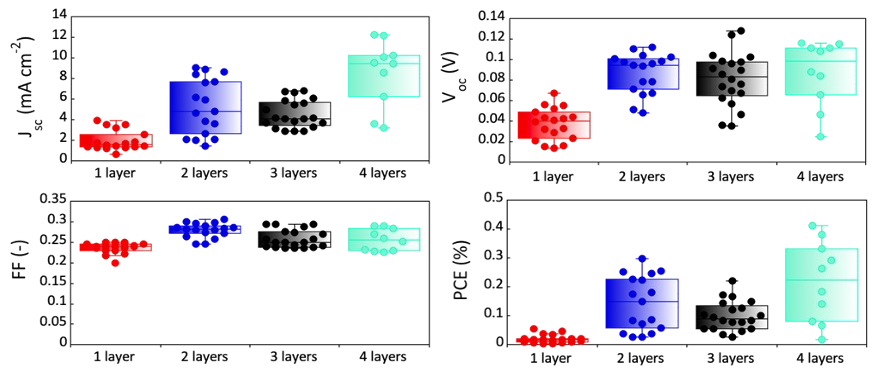


**Figure S10.** Precursor concentration variation on photovoltaic parameters of AgBiS_2_ solar cells, using *Precursor 1* (top images) and *Precursor 2* (bottom images).

**Figure S11.** Transmittance and reflectance of ITO and FTO substrates.

**Figure S12.** I-V curves of AgBiS_2_ solar cells measured at different light intensities. AgBiS_2_ film was prepared on the (a) FTO and (b) ITO substrates.

**Figure S13.** PL spectra of an AgBiS_2_ film coated on glass (AgBiS_2_/glass) and glass substrate used as background. The measurements were conducted by JASCO FP-8500 spectrofluorometer, using a laser excitation wavelength of 465 nm, with emission recorded from 500 nm to 850 nm.

**(c)**

**(b)**

**(a)**

**Figure S14**. (a) Transmittance, (b) reflectance, (c) absorptance of the FTO, FTO/SnO_2_, and FTO/SnO_2_/AgBiS_2_.

**References**

1. M. Bernechea, N. Cates, G. Xercavins, D. So, A. Stavrinadis, G. Konstantatos, *Nat. Photon.* **2016***,* 10, 521. [↑](#endnote-ref-1)
2. Y. Wang, S. R. Kavanagh, I. Burgues-Ceballos, A. Walsh, D. O. Scanlon, G. Konstantatos, *Nat. Photon.* **2022**, 16, 235. [↑](#endnote-ref-2)
3. N. Pai, J. Lu, D. C. Senevirathna, A. S. R. Chesman, T. Gengenbach, M. Chatti, U. Bach, P. C. Andews, L. Spiccia, Y. B. Chen, A. N. Simonov, *J. Mater. Chem. C* **2018**, 6, 2483. [↑](#endnote-ref-3)
4. Y. Xiao, H. Wang, F. Awai, N. Shibayama, T. Kubo, H. Segawa, *ACS Appl. Mater. Interfaces* **2022**, 14, 6994. [↑](#endnote-ref-4)
5. Y. Wu, L. Wan, W. Zhang, X. Li, J. Fang, *CrystEngComm* **2019**, 21, 3137. [↑](#endnote-ref-5)
